# GLSTNet: A Global-Local Spatial Relations and Temporal Dynamics Network for EEG-Based Emotion Recognition

**DOI:** 10.3390/bios16080421

**Published:** 2026-08-05

**Authors:** Ran Zhang, Meiyu Zhong, Caiyun Ma, Zhijun Xiao, Yuwei Zhang, Chengyu Liu

**Affiliations:** 1 School of Computer Science, Jiangsu University of Science and Technology, Zhenjiang 212003, China; 232241807233@stu.just.edu.cn (R.Z.); zhong.meiyu@just.edu.cn (M.Z.); 2 The State Key Laboratory of Bioelectronics, School of Instrument Science and Engineering, Southeast University, Nanjing 210096, China; 101300447@seu.edu.cn (C.M.); chengyu@seu.edu.cn (C.L.); 3 School of Information and Artificial Intelligence, Yangzhou University, Yangzhou 225127, China; 009043@yzu.edu.cn

**Keywords:** electroencephalography (EEG), emotion recognition, local spatial encoder (LSE), global spatial-relations encoder (GSRE), temporal dynamics encoder (TDE)

## Abstract

Electroencephalography (EEG)-based emotion recognition is an important biosensing technique for affective brain-computer interfaces (BCIs), mental-state assessment, and physiological monitoring. Existing methods often rely on a single spectral descriptor or regular two-dimensional brain maps, which makes it difficult to jointly model local spatial representations, global spatial relations, and temporal dynamics. This paper proposes GLSTNet, a global-local spatial relations and temporal dynamics network for EEG emotion recognition. EEG trials are divided into short windows, from which multi-band spectral features are extracted and arranged into compact spatial maps. The local spatial encoder (LSE) learns local spatial and spatial–spectral representations from these compact multi-band spatial maps. The global spatial-relation encoder (GSRE) models long-range spatial relations between non-adjacent electrodes using a Pearson correlation prior and a learnable residual adjacency matrix. After local and global representations are integrated through gated fusion, the temporal dynamics encoder (TDE) models consecutive EEG windows using a gated recurrent unit with temporal attention. Comprehensive validation is conducted on two public EEG emotion datasets, the Database for Emotion Analysis using Physiological Signals (DEAP) and the SJTU Emotion EEG Dataset (SEED). In the subject-dependence setting, GLSTNet achieves 93.50 ± 3.22% accuracy for valence and 93.79 ± 3.64% accuracy for arousal on DEAP, and 92.48 ± 3.30% accuracy on SEED. In the subject-independence setting with target-subject calibration, GLSTNet obtains 75.61 ± 6.19% and 79.57 ± 5.99% accuracy for DEAP valence and arousal, respectively, and 88.22 ± 4.70% accuracy on SEED. These results indicate that integrating global-local spatial relations with temporal dynamics provides an effective representation strategy for EEG-based emotion recognition.

## 1. Introduction

### 1.1. Background

Emotion shapes cognitive processes such as attention, memory, learning, and decision-making, and it is closely tied to human behavior [1]. With the development of wearable and non-invasive biosensors, affect-related physiological signals can now be recorded more conveniently in daily or semi-natural environments [2]. This sensing capability has broadened the use of emotion recognition in affective human–computer interaction [3], health-oriented monitoring, and driver-state assessment [4]. Earlier emotion recognition studies often relied on external behavioral cues, including facial expressions [5], body gestures [6], and speech signals [7]. These cues are easy to observe, but they are also affected by expression habits, intentional masking, recording conditions, and sociocultural differences. Physiological signals, by contrast, arise from neural or autonomic activity and therefore provide a more direct basis for analyzing affective states [8].

Physiological emotion recognition commonly uses two types of signals: peripheral responses and brain-related activity. Electrocardiography (ECG), electrodermal activity (EDA), and photoplethysmography (PPG) reflect autonomic or cardiovascular changes and are useful for affective monitoring. These signals, however, mainly describe bodily responses that may also vary with physical activity, respiration, and individual physiological conditions. Functional near-infrared spectroscopy (fNIRS) measures cortical hemodynamic changes and provides spatial information, but its temporal response is limited by the slower dynamics of blood-oxygen variation [9]. Electroencephalography (EEG), in contrast, records scalp-level neural electrical activity with high temporal resolution, making it well suited to rapid affective responses. For this reason, the present work focuses on EEG-based emotion recognition.

From the perspective of non-invasive biosensing, EEG emotion recognition requires models that can extract robust neural representations from low-amplitude, noisy, and subject-dependent scalp recordings. A suitable model therefore needs to support local spatial representation learning, global spatial-relation modeling, and temporal dynamics encoding across consecutive EEG windows. These requirements motivate an EEG recognition framework that jointly considers local spatial representations, global electrode relations, and temporal dynamics.

### 1.2. Related Work

Early EEG-based emotion recognition methods typically followed a feature-classifier pipeline. Lin et al. combined frequency-domain and hemispheric asymmetry features with support vector machine (SVM) classifiers for music-induced emotion recognition, showing that spectral EEG descriptors are informative for affective analysis [10]. Petrantonakis and Hadjileontiadis extracted higher-order crossing features and evaluated classical classifiers for recognizing basic emotional states from EEG [11]. Jenke et al. compared feature extraction and feature selection strategies, and their results showed that traditional pipelines are highly sensitive to the chosen features and classifiers [12]. Although these methods confirmed the value of hand-crafted EEG features, their vectorized inputs do not naturally preserve electrode layout or spatial dependency.

Convolutional neural network (CNN)-based methods were later introduced to use spatial information more directly. Li et al. transformed EEG channel features into two-dimensional maps and proposed a hierarchical CNN to learn local patterns among neighboring electrodes [13]. Compared with plain feature vectors, compact spatial maps better retain the approximate electrode layout and support local spatial representation learning. However, regular two-dimensional representations mainly encode local adjacency after electrode projection, so long-range relations between non-adjacent electrodes are still difficult to represent explicitly.

Temporal information is also important because emotional EEG responses change across consecutive segments. Zheng et al. identified stable EEG patterns over time, suggesting that temporal consistency contributes to robust emotion recognition [14]. Shen et al. proposed a four-dimensional convolutional recurrent neural network (4D-CRNN), which organized differential entropy (DE) features into a frequency–spatial–temporal structure and combined CNN-based spatial learning with recurrent modeling [15]. More recent models further strengthen temporal and spectral feature interaction. Guo et al. proposed a temporal-spectral-spatial synchronization attention network to coordinate information across temporal, spectral, and spatial dimensions [16]. Tang et al. designed an efficient-capsule network with convolutional attention to improve discriminative EEG feature learning [17]. These results supported the use of compact brain maps and temporal slices, but regular-grid convolution still provides a limited description of electrode relations beyond local neighborhoods.

Graph neural networks offer a complementary way to model EEG channels, whose relationships are not restricted to a Euclidean grid. Song et al. proposed a dynamical graph convolutional neural network (DGCNN), where electrodes are graph nodes and the adjacency matrix is learned dynamically [18]. Zhong et al. introduced regularized graph neural networks with biologically inspired constraints for cross-subject EEG emotion recognition [19]. Cheng et al. further combined dynamic graph convolution with temporal self-attention to learn channel interactions and temporal dependencies jointly [20]. Recent studies continue this direction with more flexible spatial-relation modeling. Ye et al. developed adaptive spatial–temporal aware graph learning for EEG-based emotion recognition [21]. Xiao et al. introduced a dynamical causal graph neural network to describe directed causal interactions among EEG channels [22]. Gu et al. used hierarchical multi-scale graph neural networks to capture electrode relations at different spatial scales [23]. Yan et al. proposed a spatio-temporal graph BERT network that combines graph modeling with temporal contextual learning [24]. Wang et al. further used a meta-learning wavelet graph convolutional neural network for EEG cross-subject taste classification, showing the relevance of graph learning and subject adaptation in non-invasive EEG biosensing beyond emotion recognition [25]. These graph-based methods improve the modeling of electrode relations, especially interactions that are not limited to neighboring positions, but they may underrepresent local spatial patterns provided by compact spatial maps.

Overall, prior studies show a gradual methodological progression from hand-crafted spectral features to compact spatial representation learning, temporal sequence modeling, and graph-based spatial-relation modeling. Hand-crafted feature methods are useful for describing EEG spectral responses, but they are weak in preserving spatial organization. CNN-based methods learn local spatial patterns from two-dimensional representations, but they do not explicitly model non-adjacent electrode relations. Graph-based methods describe electrode interactions more naturally, yet they can benefit from local map-based spatial features. Temporal models are useful for dynamic affective responses, but their effectiveness depends on robust spatial and spectral front-end representations. These observations motivate a unified framework that combines local spatial representation learning, global spatial-relation modeling, and temporal dynamics encoding.

### 1.3. The Contributions of This Study

This study proposes GLSTNet, a global-local spatial relations and temporal dynamics network for EEG-based emotion recognition. The framework first extracts multi-band spectral descriptors from short EEG windows and arranges electrode features as compact spatial maps. The local spatial encoder (LSE) learns local spatial and spatial–spectral representations from compact multi-band spatial maps, while the global spatial-relation encoder (GSRE) constructs an adaptive electrode graph to model long-range relations between non-adjacent electrodes. After local and global representations are integrated through gated fusion, the temporal dynamics encoder (TDE) uses a gated recurrent unit (GRU) with temporal attention to encode consecutive EEG windows for sequence-level classification.

The main contributions of this study are summarized as follows:

1. **A global-local spatial modeling strategy is proposed to integrate compact spatial maps and adaptive electrode relations.** The proposed framework organizes multi-band EEG descriptors into compact two-dimensional spatial representations and uses the LSE to capture local spatial and spatial–spectral variations among neighboring electrode regions. To complement this local representation, the GSRE constructs an adaptive electrode graph by combining a Pearson correlation prior with a learnable residual adjacency matrix, allowing GLSTNet to model long-range spatial relations between non-adjacent electrodes.
2. **A temporal dynamics encoder is developed to capture emotion-related changes across consecutive EEG windows.** The LSE and GSRE representations are adaptively integrated through gated fusion at each time slice, and the TDE further encodes temporal dynamics using a GRU with temporal attention. This design enables GLSTNet to combine local spatial patterns, global electrode relations, and emotionally informative temporal segments within a unified classification framework.
3. **Generalization ability is validated on multi-source EEG emotion datasets.** The proposed framework is evaluated on DEAP and SEED under subject-dependence and subject-independence settings, covering binary valence and arousal recognition and three-class emotion recognition. The accuracy and F1 results show that GLSTNet maintains stable and competitive performance across different dataset characteristics, label spaces, and evaluation settings.

## 2. Materials and Methods

**Figure 1:**
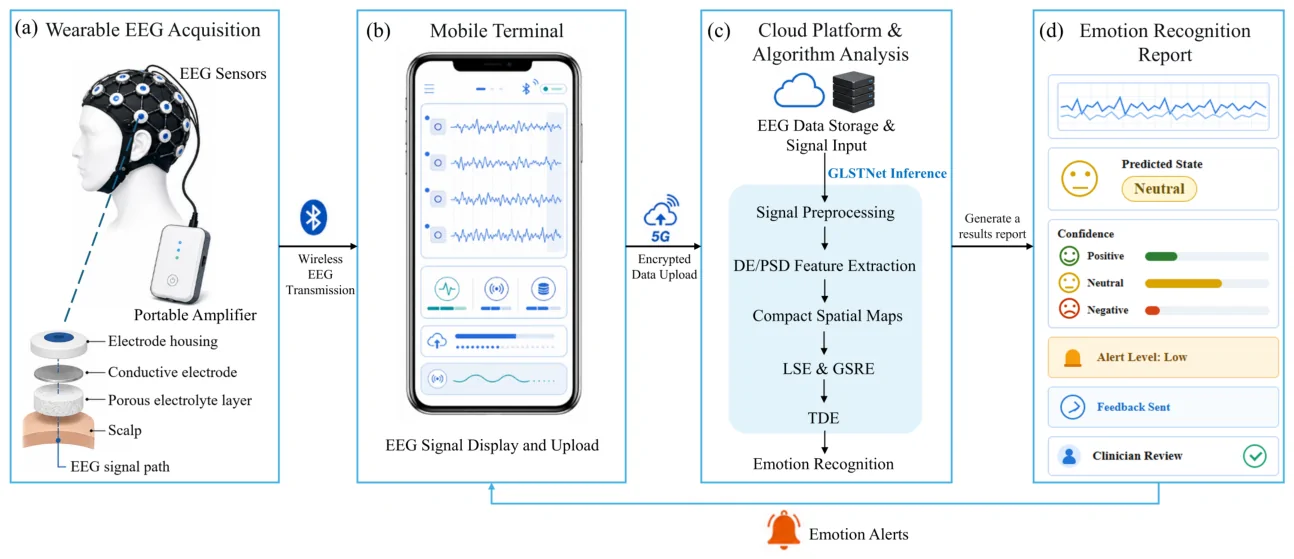
Workflow of the EEG-based emotion recognition system, including wearable EEG acquisition, mobile terminal display and upload, cloud-side GLSTNet analysis, and report generation with feedback alerts.

Figure 1 presents the workflow of the EEG-based emotion recognition system, including wearable EEG acquisition, mobile terminal display and upload, cloud-side GLSTNet analysis, and report generation with feedback alerts. In this workflow, scalp EEG signals are acquired by wearable sensors and a portable amplifier, displayed on a mobile terminal for inspection, and uploaded to a cloud platform. The cloud-side module organizes the uploaded signals into spectral features and compact spatial representations, after which GLSTNet performs emotion recognition through the LSE, GSRE, and TDE. The recognition output is then returned to a remote monitoring interface for emotion-state feedback or alerts. Based on this application context, the following sections describe the datasets, feature construction procedure, spatial mapping strategy, and GLSTNet architecture.

### 2.1. Datasets

**Figure 2:**
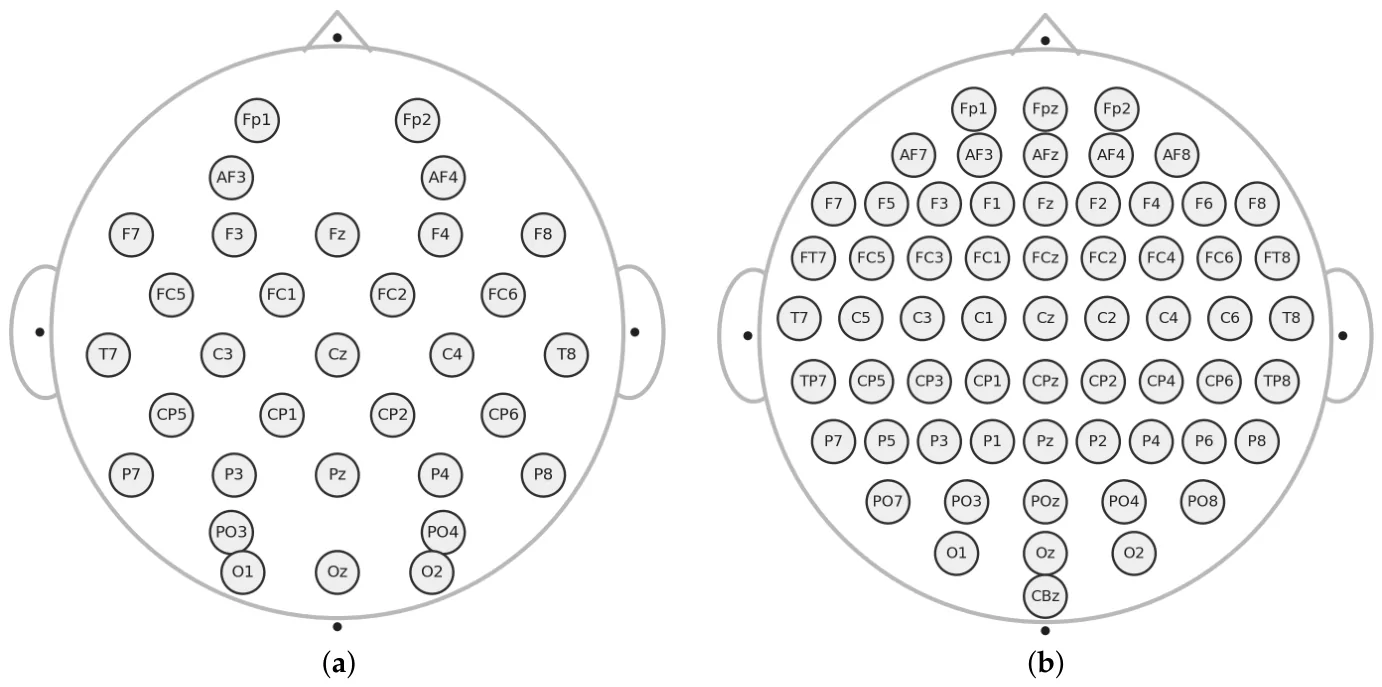
EEG channel distributions of the two datasets. (**a**) DEAP 32-channel distribution. (**b**) SEED 62-channel distribution.

Experiments are conducted on two public EEG emotion recognition datasets: the Database for Emotion Analysis using Physiological Signals (DEAP) [26] and the SJTU Emotion EEG Dataset (SEED) [27]. DEAP is used for binary classification along the valence and arousal dimensions, whereas SEED is used for three-class emotion recognition. Because the two datasets differ in subject number, channel configuration, sampling rate, stimuli, and label definition, they provide complementary settings for evaluating the proposed framework. Figure 2 displays the EEG channel distributions of the two datasets, including the DEAP 32-channel distribution and the SEED 62-channel distribution.

The DEAP dataset contains recordings from 32 participants watching 40 one-minute music videos. This study uses only the EEG signals, which include 32 channels and are sampled at 128 Hz in the preprocessed version. Each trial is annotated with valence, arousal, dominance, and liking scores. Following common settings, valence and arousal are treated as two independent binary classification tasks, where scores higher than 5 are assigned to the high-emotion class and the remaining scores are assigned to the low-emotion class.

The SEED dataset contains EEG recordings from 15 participants watching emotion-eliciting film clips. Each participant completed three sessions, and each session contains 15 trials corresponding to positive, neutral, and negative emotions. The preprocessed EEG signals contain 62 channels and are sampled at 200 Hz. In this study, SEED is used for three-class emotion recognition. Table 1 summarizes the DEAP and SEED datasets used in this work.

### 2.2. Proposed GLSTNet Architecture

**Figure 3:**
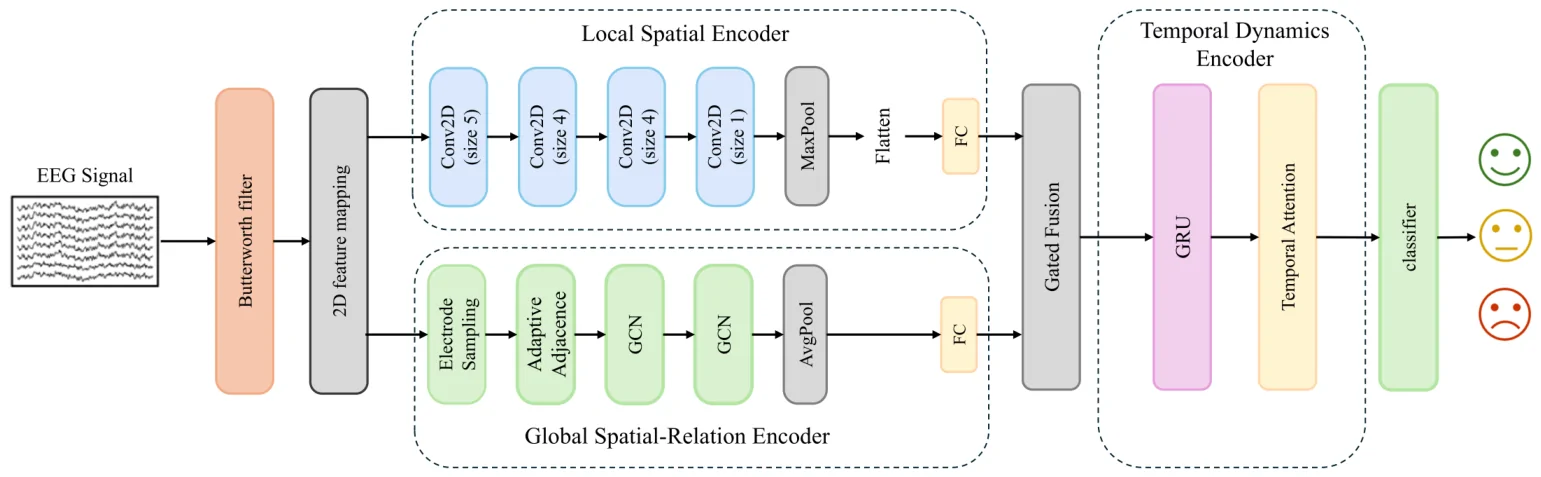
Overall architecture of the proposed GLSTNet framework.

The proposed GLSTNet framework contains the LSE for local spatial representation learning, the GSRE for global spatial-relation modeling, a gated fusion mechanism, and the TDE for temporal dynamics encoding. Figure 3 depicts the overall architecture of the proposed GLSTNet framework. The model first extracts spectral features from short EEG windows and arranges them as compact multi-band spatial maps. For each time slice, the LSE learns local spatial and spatial–spectral representations from these maps. In parallel, the GSRE samples the valid electrode positions from the same map and performs graph convolution to model long-range spatial relations between non-adjacent electrodes. The gated fusion mechanism adaptively combines the LSE and GSRE representations, and the TDE uses a gated recurrent unit (GRU) with temporal attention to encode consecutive windows for classification.

#### 2.2.1. Spectral Feature Extraction

**Figure 4:**
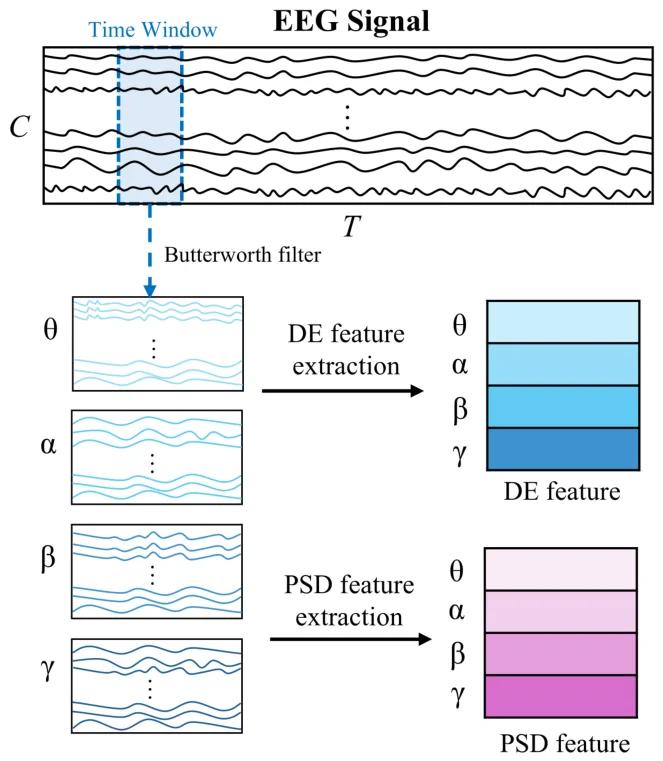
Spectral feature extraction from EEG time windows.

For an EEG trial with *C* channels and *T* sampling points, the signal is segmented into non-overlapping 0.5 s windows, which have been shown to balance temporal resolution with reliable DE and PSD estimation [28]. Given the sampling rate $F_{s}$ and the window length Δt, each window contains:

(1) $$L=F_{s}\Delta t,$$

where *L* is the number of sampling points in one window. The trial is then divided into *M* windows, and the *m*-th window is written as $W_{m}\in R^{C\times L}$, where m=1,2,…,M. Figure 4 illustrates the spectral feature extraction from EEG time windows.

A third-order Butterworth band-pass filter is applied to each window to obtain the theta (4–8 Hz), alpha (8–14 Hz), beta (14–31 Hz), and gamma (31–51 Hz) bands. For the *i*-th electrode, the *m*-th window, and the *k*-th frequency band, the filtered segment is represented by $s_{i,m}^{\left(k\right)}$, and its variance is represented by $\sigma_{i,m,k}^{2}$. The same calculation is repeated for every channel, time window, and frequency band.

Differential entropy (DE) is used to describe the complexity of the band-limited EEG signal [29]. Assuming that a filtered EEG segment approximately follows a Gaussian distribution, the DE feature of the *i*-th electrode, the *m*-th window, and the *k*-th band is calculated as:

(2) $${\mathrm{DE}}_{i,m}^{\left(k\right)}=\frac{1}{2}\log\left(2\pi e\sigma_{i,m,k}^{2}\right),$$

where $\sigma_{i,m,k}^{2}$ is the variance of $s_{i,m}^{\left(k\right)}$. The resulting DE value is stored as one band-wise feature of the corresponding electrode and window.

Power spectral density (PSD) is extracted as a complementary power-related feature [30]. Let $P_{i,m}^{\left(k\right)}\left(f\right)$ be the power spectrum of $s_{i,m}^{\left(k\right)}$ at frequency *f*. The band-wise PSD feature is computed as the average spectral power within the selected frequency band:

(3) $${\mathrm{PSD}}_{i,m}^{\left(k\right)}=\frac{1}{|\Omega_{k}|}\sum_{f\in \Omega_{k}}P_{i,m}^{\left(k\right)}\left(f\right),$$

where $\Omega_{k}$ is the set of frequency bins in the *k*-th band and $|\Omega_{k}|$ is the number of bins in this band. As with DE, PSD is calculated for each electrode, window, and frequency band.

For each electrode, the selected spectral descriptors are organized into the feature vector $e_{i,m}$. In the DE+PSD setting, $e_{i,m}$ contains the *K* DE values followed by the *K* PSD values, so $e_{i,m}\in R^{2K}$. When only DE is used, $e_{i,m}$ contains the *K* DE values and $e_{i,m}\in R^{K}$. The feature vectors of all *C* electrodes are then stacked by electrode order to form $E_{m}\in R^{C\times D}$, where each row corresponds to one electrode and each column corresponds to one feature dimension. For DEAP, the baseline segment is used to reduce trial-level bias by subtracting the average baseline feature matrix from the trial features. For SEED, the preprocessed trial signals are directly used for feature extraction.

#### 2.2.2. Two-Dimensional Spatial Mapping

The feature matrix $E_{m}$ is ordered by electrode index, which is convenient for storage but weakly reflects scalp neighborhood structure. To obtain a compact spatial input for convolutional learning, this work adopts the 8×9 two-dimensional spatial mapping strategy used in previous EEG emotion recognition studies [15]. Electrode-wise features are projected onto a regular grid according to electrode positions, and empty grid locations are filled with zeros.

**Figure 5:**
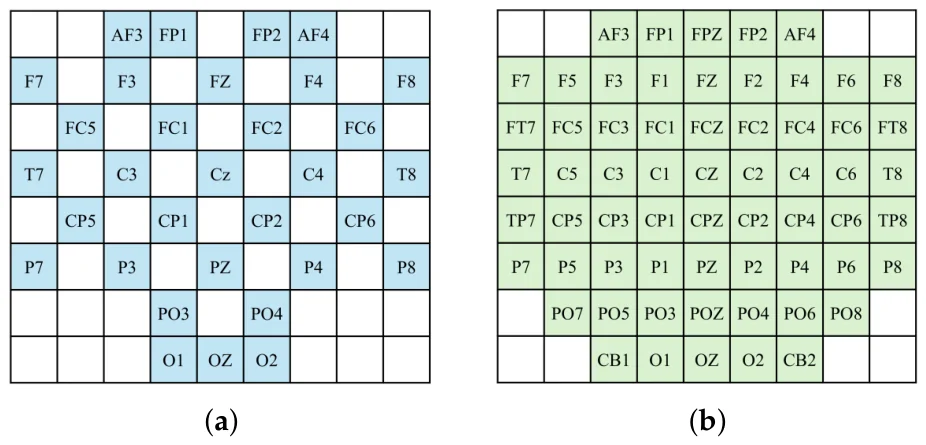
Dataset-specific 8×9 two-dimensional spatial mappings. (**a**) DEAP 32-channel mapping. (**b**) SEED 62-channel mapping.

DEAP and SEED use different electrode-to-grid mappings because their channel configurations are different. The mapping functions are written as $\pi_{\mathrm{DEAP}}$ and $\pi_{\mathrm{SEED}}$, respectively. Both datasets are represented on an 8×9 grid, but the valid electrode positions differ: DEAP contains 32 EEG channels, whereas SEED contains 62. Figure 5 presents the dataset-specific 8×9 two-dimensional spatial mappings for DEAP and SEED.

**Figure 6:**
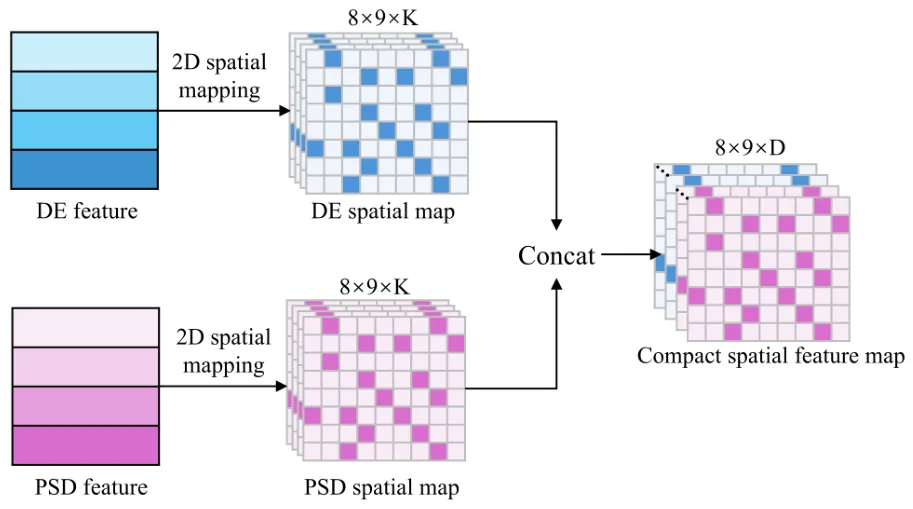
Construction of the multi-channel 2D feature map.

After the dataset-specific electrode positions are determined, each selected spectral descriptor is projected onto the 8×9 grid. For the DE+PSD configuration, the DE feature matrix and the PSD feature matrix of the *m*-th window are first arranged as two spatial maps, $X_{m}^{\mathrm{DE}}\in R^{8\times 9\times K}$ and $X_{m}^{\mathrm{PSD}}\in R^{8\times 9\times K}$, using the mapping function π. These two maps are then concatenated along the feature dimension to form $X_{m}\in R^{8\times 9\times D}$, where *D* corresponds to the combined DE and PSD feature dimension. For a DE-only configuration, $X_{m}$ is the DE spatial map with *K* feature channels. Figure 6 depicts the construction of the multi-channel 2D feature map. In the DE+PSD configuration, the two descriptors provide complementary band-wise descriptions of the same EEG window, while the 8×9 arrangement preserves the approximate spatial organization of the electrodes.

After mapping, *Q* consecutive spatial maps are grouped as one model input $X\in R^{Q\times 8\times 9\times D}$. In this sequence, $X_{q}$ is the *q*-th spatial slice, and *Q* is the number of slices sent to the TDE.

#### 2.2.3. Local Spatial Encoder (LSE)

**Figure 7:**
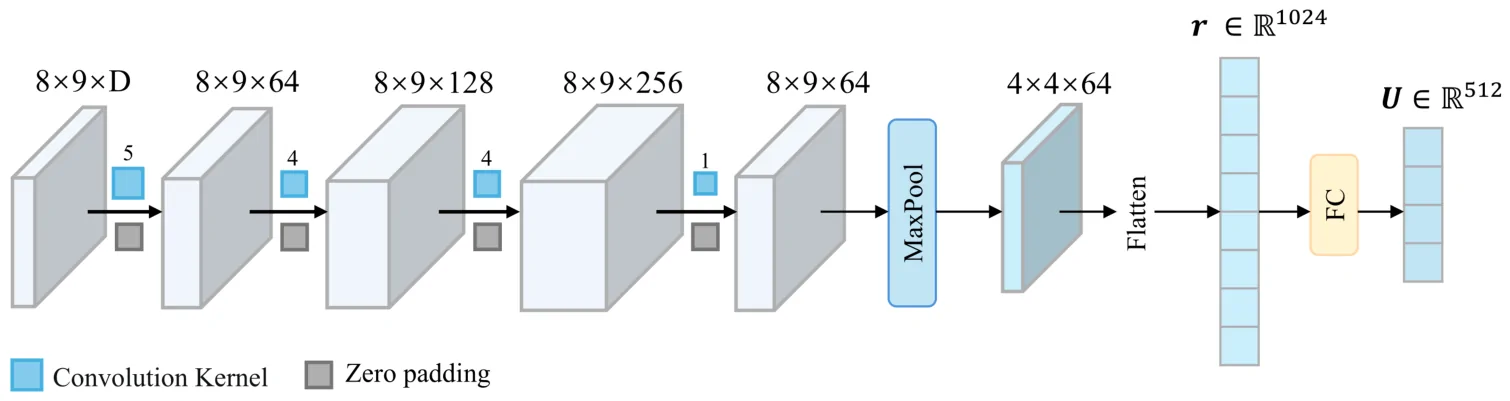
Structure of the local spatial encoder (LSE).

The LSE learns local spatial and spatial–spectral representations from compact multi-band spatial maps. The structure of the local spatial encoder (LSE) is presented in Figure 7. In this module, each time slice $X_{q}\in R^{8\times 9\times D}$ passes through four convolutional layers. The first 5×5 convolution covers a relatively broad local scalp region, and the following two 4×4 convolutions refine local spatial and spatial–spectral combinations among neighboring electrode regions. The final 1×1 convolution performs channel projection, reducing the feature maps from 256 to 64 without changing the spatial size.

With same padding and a stride of 1, the convolutional part preserves the 8×9 spatial resolution, while the feature-channel dimension changes from *D* to 64, 128, 256, and finally 64. A 2×2 max-pooling layer then reduces the spatial size to 4×4:

(4) $$F_{q}^{\mathrm{LSE}}=\mathrm{MaxPool}\left(\mathrm{Conv}\left(X_{q}\right)\right),$$

where $F_{q}^{\mathrm{LSE}}\in R^{4\times 4\times 64}$ is the pooled LSE feature tensor of the *q*-th time slice, and Conv(·) represents the four convolutional layers in the LSE. The pooled tensor is flattened into a vector:

(5) $$r_{q}=\mathrm{Flatten}\left(F_{q}^{\mathrm{LSE}}\right),$$

where $r_{q}\in R^{1024}$ is the flattened local feature vector and 1024=4×4×64. A fully connected layer projects it into a 512-dimensional LSE representation:

(6) $$U_{q}=\delta \left(W_{U}r_{q}+b_{U}\right),$$

where δ(·) is the rectified linear unit activation, $W_{U}$ and $b_{U}$ are the weight matrix and bias of the fully connected layer, and $U_{q}\in R^{512}$ represents the local spatial and spatial–spectral information of the *q*-th time slice.

#### 2.2.4. Global Spatial-Relation Encoder (GSRE)

**Figure 8:**
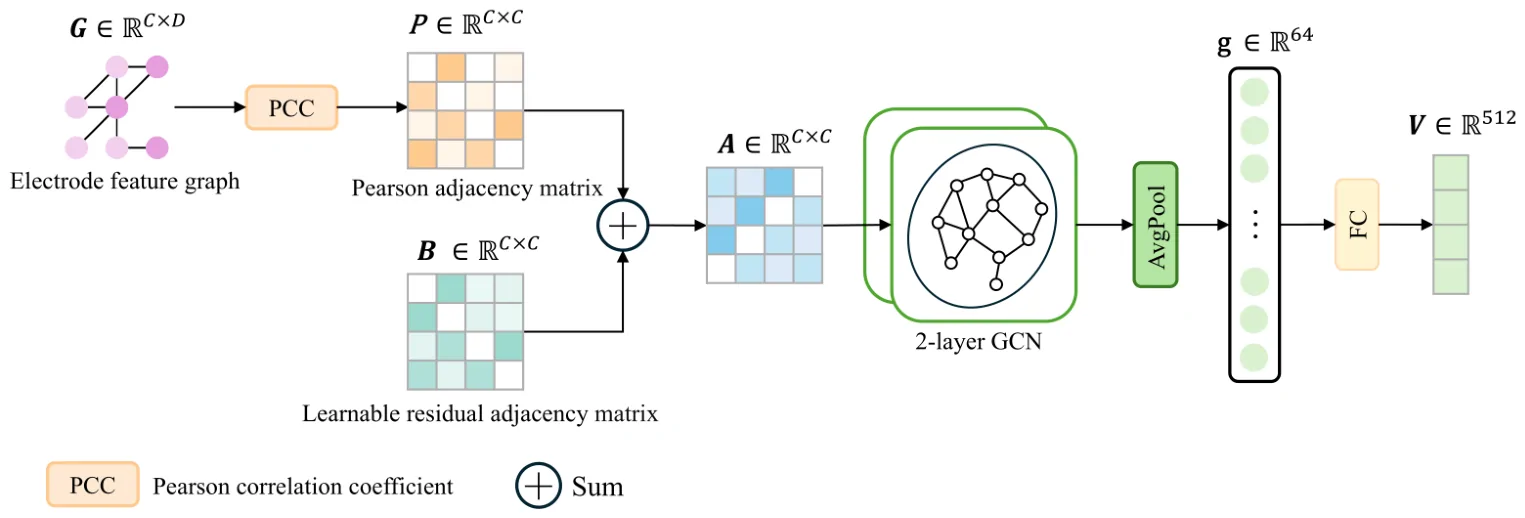
Structure of the global spatial-relation encoder (GSRE).

The LSE captures local spatial and spatial–spectral representations from compact multi-band spatial maps, whereas emotion-related EEG activity may also involve interactions between distant electrodes. The GSRE models these non-local relationships by treating electrodes as graph nodes. The structure of the global spatial-relation encoder (GSRE) is given in Figure 8.

For the *q*-th time slice, the real electrode positions are sampled from the two-dimensional map according to the mapping π, yielding the node feature matrix:

(7) $$G_{q}=\mathrm{Sample}\left(X_{q},\pi \right).$$

The matrix $G_{q}\in R^{C\times D}$ contains the sampled node features. Each row corresponds to one EEG electrode, and each column corresponds to one selected spectral feature dimension. The GSRE therefore does not use the entire 8×9 grid as the graph; only valid electrode positions are used as nodes.

A Pearson adjacency matrix is calculated from the training set as a functional connectivity prior. The prior adjacency matrix is defined element-wise as:

(8) $$P_{ij}=\left|\frac{\sum_{r=1}^{R}\left(o_{i,r}-{\bar{o}}_{i}\right)\left(o_{j,r}-{\bar{o}}_{j}\right)}{\sqrt{\sum_{r=1}^{R}{\left(o_{i,r}-{\bar{o}}_{i}\right)}^{2}}\sqrt{\sum_{r=1}^{R}{\left(o_{j,r}-{\bar{o}}_{j}\right)}^{2}}+\epsilon }\right|,$$

where $P_{ij}$ is the (i,j) element of the Pearson prior adjacency matrix $P\in R^{C\times C}$. Here, P is estimated only from the training set, *R* is the number of training observations used to estimate this prior, $o_{i,r}$ is a scalar electrode-level value for electrode *i* in the *r*-th training observation, ${\bar{o}}_{i}$ is the mean of $o_{i,r}$ over all *R* training observations, and ϵ is a small constant used to avoid division by zero. When the selected electrode feature is a *D*-dimensional spectral feature vector, $o_{i,r}$ is obtained by averaging the *D* feature dimensions of electrode *i* in the *r*-th training observation. Because P is estimated from training observations only, test samples are not involved in the prior construction, which avoids test-set information leakage. The absolute value is used because both positive and negative correlations may indicate functional coupling between electrodes.

To avoid relying on a fixed prior graph, a learnable residual adjacency matrix $B\in R^{C\times C}$ is introduced. The adjacency matrix used for graph convolution is:

(9) A=ReLU(P+B)+I,

where I is the identity matrix. The Pearson matrix P provides the initial functional connectivity prior, and B is updated by backpropagation during training. In this way, the graph structure is allowed to adapt to the emotion classification objective.

Before graph convolution, the adjacency matrix is row-normalized:

(10) $$\tilde{A}=D_{A}^{-1}A,\;D_{A}=\mathrm{diag}\left(d_{1},\ldots ,d_{C}\right),\;d_{i}=\sum_{j=1}^{C}A_{ij},$$

where $\tilde{A}$ is the normalized adjacency matrix, $D_{A}$ is the degree matrix of A, $A_{ij}$ is the (i,j) element of A, and $d_{i}$ is the degree value of electrode node *i*. Two graph convolutional layers are then applied:

(11) $$N_{q}^{\left(1\right)}=\delta \left(\tilde{A}G_{q}W_{1}+b_{1}\right),$$

where $N_{q}^{\left(1\right)}$ is the hidden node representation after the first graph convolutional layer, $W_{1}$ is the trainable graph convolution weight matrix, and $b_{1}$ is the bias term.

(12) $$N_{q}=\delta \left(\tilde{A}N_{q}^{\left(1\right)}W_{2}+b_{2}\right),$$

where $N_{q}\in R^{C\times 64}$ is the final node representation of the *q*-th time slice, while $W_{2}$ and $b_{2}$ are the parameters of the second graph convolution. The output $N_{q}$ contains updated electrode representations after aggregating information from functionally connected electrodes. Let $n_{q,i}\in R^{64}$ represent the graph-convolution feature vector of electrode *i* in the *q*-th time slice. Global average pooling is then used to obtain a graph-level representation:

(13) $$g_{q}=\frac{1}{C}\sum_{i=1}^{C}n_{q,i},$$

where $g_{q}\in R^{64}$ summarizes the electrode-level graph features in the *q*-th time slice. Finally, a fully connected layer maps $g_{q}$ into a 512-dimensional GSRE representation:

(14) $$V_{q}=\delta \left(W_{V}g_{q}+b_{V}\right),$$

where $g_{q}$ is the graph-level representation after average pooling, $W_{V}$ and $b_{V}$ are the parameters of the fully connected projection, and $V_{q}\in R^{512}$ is the global spatial-relation representation of the *q*-th time slice.

#### 2.2.5. Gated Fusion Mechanism

For each time slice, the LSE outputs $U_{q}\in R^{512}$, and the GSRE outputs $V_{q}\in R^{512}$. The two vectors describe different aspects of the same EEG segment: $U_{q}$ emphasizes local spatial and spatial–spectral representations, whereas $V_{q}$ emphasizes long-range spatial relations and functional interactions among electrodes. A gated fusion mechanism is used instead of direct concatenation or averaging so that the contribution of each representation can vary across feature dimensions.

The gate vector is computed as:

(15) $$\lambda_{q}=\sigma \left(W_{\lambda }\left[U_{q};V_{q}\right]+b_{\lambda }\right),$$

where $\lambda_{q}\in R^{512}$ is the gate vector for the *q*-th time slice, [·;·] means vector concatenation, σ(·) is the sigmoid function, and $W_{\lambda }$ and $b_{\lambda }$ are trainable fusion parameters. The fused feature is:

(16) $$Z_{q}=\lambda_{q}⊙U_{q}+\left(1-\lambda_{q}\right)⊙V_{q},$$

where $Z_{q}\in R^{512}$ is the fused feature of the *q*-th time slice and ⊙ is element-wise multiplication. A larger value of $\lambda_{q}$ increases the contribution of the LSE representation, while a smaller value gives more weight to the GSRE representation. The fused features from *Q* consecutive time slices form the temporal feature sequence $Z\in R^{Q\times 512}$.

#### 2.2.6. Temporal Dynamics Encoder (TDE)

**Figure 9:**
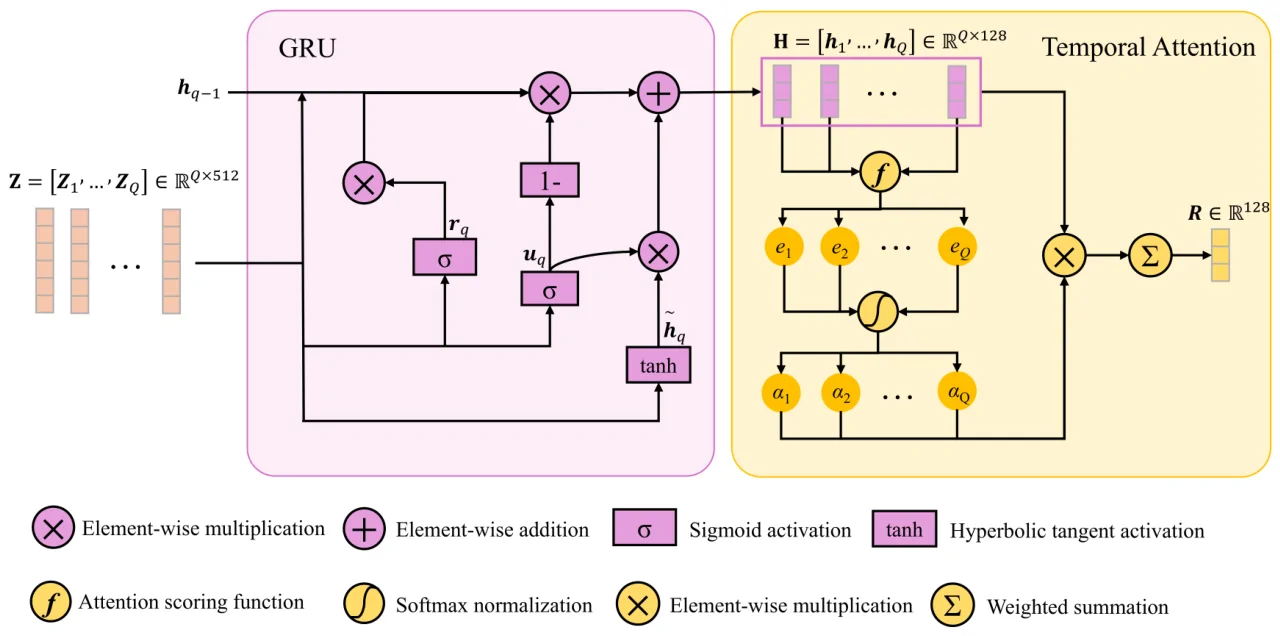
Structure of the temporal dynamics encoder (TDE).

EEG emotion responses are temporally dynamic, and a single 0.5 s window may be insufficient for stable recognition. The TDE therefore models dependencies among *Q* consecutive fused features. Figure 9 illustrates the structure of the temporal dynamics encoder (TDE), which contains one GRU layer and a temporal attention mechanism.

The GRU receives the fused feature sequence $Z\in R^{Q\times 512}$. The same GRU is recurrently applied across the *Q* time slices, rather than using *Q* independent GRUs. For the *q*-th time slice, the update process is formulated as:

(17) $$\mu_{q}=\sigma \left(W_{\mu }Z_{q}+U_{\mu }h_{q-1}+b_{\mu }\right),$$

(18) $$\rho_{q}=\sigma \left(W_{\rho }Z_{q}+U_{\rho }h_{q-1}+b_{\rho }\right),$$

(19) $${\tilde{h}}_{q}=\tanh\left(W_{h}Z_{q}+U_{h}\left(\rho_{q}⊙h_{q-1}\right)+b_{h}\right),$$

(20) $$h_{q}=\left(1-\mu_{q}\right)⊙h_{q-1}+\mu_{q}⊙{\tilde{h}}_{q},$$

where $\mu_{q}$ is the update gate, $\rho_{q}$ is the reset gate, ${\tilde{h}}_{q}$ is the candidate hidden state, and $h_{q}\in R^{128}$ is the GRU hidden state. The matrices $W_{\mu }$, $W_{\rho }$, and $W_{h}$ act on the current input $Z_{q}$; $U_{\mu }$, $U_{\rho }$, and $U_{h}$ act on the previous hidden state $h_{q-1}$; and the corresponding b terms are trainable biases. The hidden states generated from all *Q* slices form the GRU output sequence $H\in R^{Q\times 128}$. Temporal attention is used to assign different weights to different time slices. The attention score is calculated as:

(21) $$e_{q}=v_{a}^{⊤}\tanh\left(W_{a}h_{q}+b_{a}\right),$$

and the normalized attention weight is:

(22) $$\alpha_{q}=\frac{\exp(e_{q})}{\sum_{r=1}^{Q}\exp\left(e_{r}\right)},$$

where $e_{q}$ is the attention score of the *q*-th time slice, $v_{a}$, $W_{a}$, and $b_{a}$ are trainable attention parameters, $\alpha_{q}$ is the normalized attention weight assigned to this slice, and *r* is the index used to sum over all *Q* time slices. The final temporal representation is obtained by weighted summation:

(23) $$R=\sum_{q=1}^{Q}\alpha_{q}h_{q}.$$

The vector $R\in R^{128}$ is the final temporal representation used by the classifier. The emotion class probability is predicted by a fully connected layer followed by Softmax:

(24) $$p=\mathrm{Softmax}(W_{c}R+b_{c}),$$

where $p\in R^{J}$ is the predicted class-probability vector, and $W_{c}$ and $b_{c}$ are classifier parameters. The final predicted label is obtained by selecting the class with the largest probability, written as $\hat{y}=\arg\max_{j}p_{j}$.

### 2.3. Training Objective and Evaluation Metrics

The network is trained by minimizing the cross-entropy loss. For a mini-batch with $N_{b}$ samples and *J* emotion classes, the loss is:

(25) $$L=-\frac{1}{N_{b}}\sum_{i=1}^{N_{b}}\sum_{j=1}^{J}y_{ij}\log\left(p_{ij}\right),$$

where $y_{ij}$ is the one-hot label element indicating whether sample *i* belongs to class *j*, and $p_{ij}$ is the predicted probability of class *j* for this sample. All trainable parameters, including convolutional parameters, graph convolutional parameters, the residual adjacency matrix B, fusion parameters, GRU parameters, attention parameters, and classifier parameters, are optimized jointly.

Accuracy and F1 are used as the evaluation metrics. Accuracy is defined at the sample level as:

(26) $$\mathrm{Accuracy}=\frac{1}{N}\sum_{i=1}^{N}I\left({\hat{y}}_{i}=y_{i}\right),$$

where *N* is the number of test samples, $y_{i}$ is the ground-truth label of the *i*-th sample, ${\hat{y}}_{i}$ is its predicted label, and I(·) is the indicator function, which equals 1 for a correct prediction and 0 otherwise.

For each emotion class, precision, recall, and F1 are computed as follows:

(27) $$\begin{array}{cc} \mathrm{Precision} & =\frac{TP}{TP+FP},\\ \mathrm{Recall} & =\frac{TP}{TP+FN}, \end{array},$$

where TP, FP, and FN denote the true positives, false positives, and false negatives for the currently considered class, respectively. F1 is then calculated as:

(28) $$F1=\frac{2\times \mathrm{Precision}\times \mathrm{Recall}}{\mathrm{Precision}+\mathrm{Recall}}.$$

The F1 reported in this paper is the average of the class-wise F1 values. For SEED, it is averaged over the negative, neutral, and positive classes; for DEAP, it is averaged over the low- and high-emotion classes.

### 2.4. Algorithm Procedure

Algorithm 1 summarizes the training and prediction procedure of the proposed GLSTNet framework.
**Algorithm 1** Training and prediction procedure of the proposed GLSTNet framework**Require:** EEG trials S, labels Y, selected frequency bands, mapping function π, sequence length *Q*, epochs *E***Ensure:** Predicted labels $\hat{Y}$  1:Let M denote the GLSTNet model.  2:Segment each EEG trial into 0.5 s windows according to Equation (1).  3:Extract the selected DE and/or PSD features according to Equations (2) and (3).  4:Stack electrode-wise features to construct $E_{m}$.  5:Map $E_{m}$ into the compact spatial map $X_{m}$ using π.  6:Group *Q* consecutive maps into the model input X.  7:Compute the Pearson prior P from the training data by Equation (8) and initialize B.  8:**for** epoch =1 to *E* **do**  9:    **for** each mini-batch **do**10:        **for** q=1 to *Q* **do**11:           Obtain the LSE representation $U_{q}$ by Equations (4)–(6).12:           Sample node features $G_{q}$ by Equation (7).13:           Construct and normalize A, and obtain the GSRE representation $V_{q}$ by Equations (9)–(14).14:           Fuse $U_{q}$ and $V_{q}$ into $Z_{q}$ by Equations (15) and (16).15:        **end for**16:        Form the temporal feature sequence Z and encode temporal dynamics with the TDE by Equations (17)–(23).17:        Predict class probabilities p by Equation (24).18:        Update M by minimizing Equation (25).19:    **end for**20:**end for**21:Predict class probabilities p for each test sample using the trained M.22:Obtain predicted labels $\hat{Y}$ by selecting the class with the highest probability.23:**return** $\hat{Y}$

## 3. Results

### 3.1. Experimental Settings

All experiments are implemented in PyTorch 2.6.0 on a workstation with an NVIDIA GeForce RTX 4090 GPU (NVIDIA Corporation, Santa Clara, CA, USA). The Adam optimizer is used with an initial learning rate of 0.001, and the maximum number of training epochs is set to 100. The mini-batch size is set to $N_{b}=64$ and gradient clipping with a maximum norm of 5.0 is applied. DEAP uses DE+PSD as the main feature configuration, whereas SEED uses DE as the main feature configuration. The input sequence length is set to Q=6 in the main experiments. Feature standardization is performed using the statistics of the training set, and the same statistics are applied to the test set. Accuracy and F1 are used as the evaluation metrics, and all metric values are reported as percentages.

For subject-dependence evaluation, stratified five-fold cross-validation is conducted separately for each subject, following a commonly used protocol in EEG-based emotion recognition. The reported subject-dependent results are averaged over all subjects and folds. For subject-independence evaluation, one subject is selected as the target subject in each round, while the remaining subjects are used as source subjects for model training. A small labeled subset of the target subject is used for target-subject calibration, and the remaining target-subject samples are reserved for testing. The calibration ratio is set to 20%, with 10 calibration epochs and a calibration learning rate of 0.0001. This protocol evaluates whether the model can adapt to an unseen subject when limited target-subject calibration data are available. The following sections report the results separately on DEAP and SEED so that the dataset-specific behavior can be analyzed more clearly.

### 3.2. Experiment on DEAP

Under subject-dependence evaluation, GLSTNet achieves 93.50 ± 3.22% accuracy and 93.18 ± 3.36% F1 for valence classification, and 93.79 ± 3.64% accuracy and 92.88 ± 3.71% F1 for arousal classification on DEAP. The following subject-level tables further examine whether the averaged performance is supported by consistent participant-wise behavior.

Table 2 reports the subject-dependent accuracy on the DEAP dataset for valence and arousal classification. The subject-dependent accuracy table indicates that most subjects obtain high accuracy in both affective dimensions. The mean values are close to the median values of 94.50% for valence and 94.94% for arousal, indicating that the averaged performance is not dominated by only a few easily classified subjects. Arousal is slightly higher than valence, which may be related to the stronger association between arousal and physiological activation intensity.

**Figure 10:**
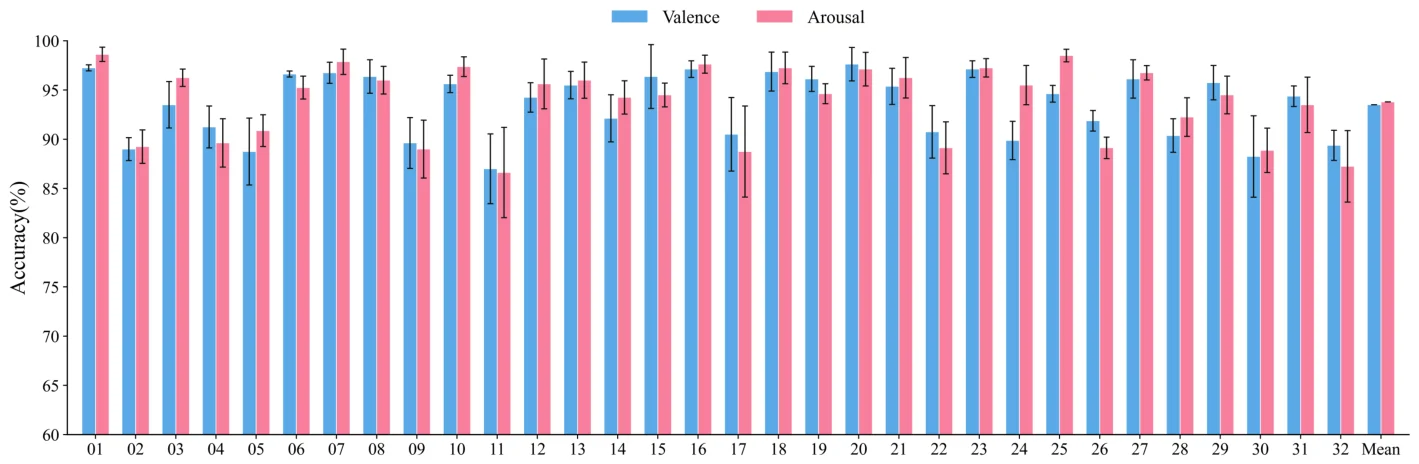
Subject-dependent accuracy of valence and arousal classification on the DEAP dataset.

Figure 10 plots the subject-dependent accuracy of valence and arousal classification on the DEAP dataset. The visual trend is consistent with the subject-dependent accuracy in Table 2: valence and arousal remain high for most subjects, while several subjects show relatively lower values in both dimensions. This distribution indicates that the main variation comes from participant-level differences rather than from an isolated failure on a single affective dimension.

Presented in Table 3 is the subject-dependent F1 on the DEAP dataset for valence and arousal classification. The F1 values remain close to the corresponding accuracy values for both valence and arousal, indicating that the model does not obtain high accuracy by favoring one class. This agreement is consistent with the subject-dependent accuracy pattern in Table 2.

**Figure 11:**
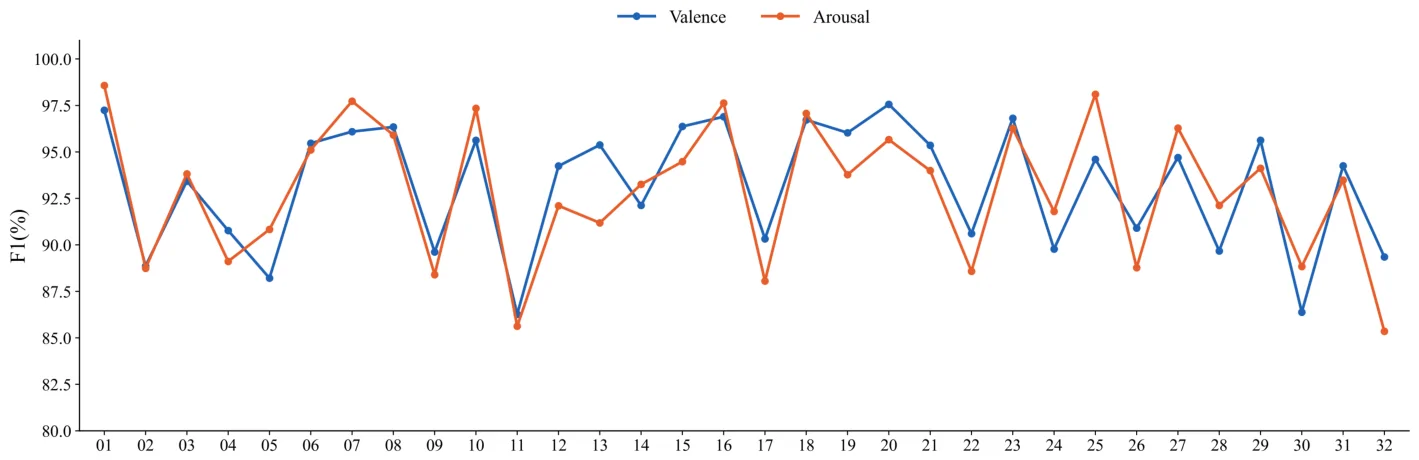
Subject-dependent F1 of valence and arousal classification on the DEAP dataset.

Figure 11 plots the subject-dependent F1 of valence and arousal classification on the DEAP dataset. The F1 curves follow the accuracy curves closely across subjects. This consistency further suggests balanced recognition between the two classes in each binary task, rather than a performance pattern driven by class imbalance.

**Figure 12:**
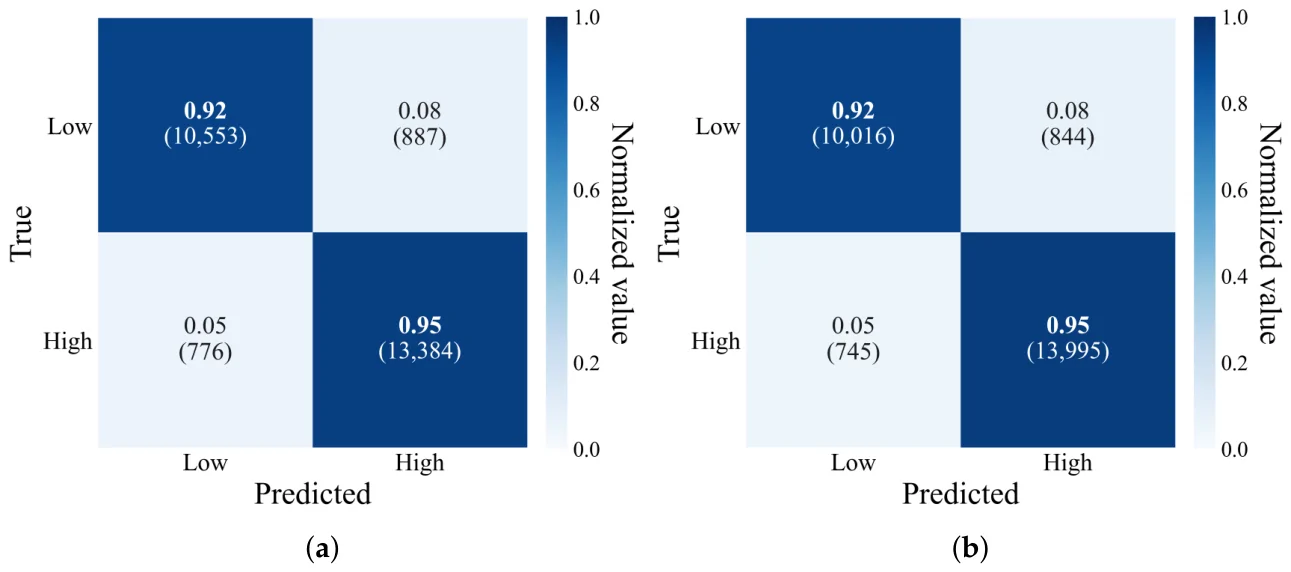
Confusion matrices on the DEAP dataset. (**a**) Valence classification. (**b**) Arousal classification.

Figure 12 presents the confusion matrices on the DEAP dataset for valence and arousal classification. For valence, the recall values of the low- and high-valence classes are 92.00% and 95.00%, respectively. For arousal, the recall values of the low- and high-arousal classes are also 92.00% and 95.00%, respectively. The errors are relatively balanced between the two classes, while high-level emotional states are slightly easier to identify. This result is consistent with the participant-wise accuracy and F1 trends.

The subject-independence results on DEAP further evaluate whether the learned representations can be adapted to a target participant who is not included in source-subject training, with only limited target-subject calibration data available. In this setting, GLSTNet obtains 75.61 ± 6.19% accuracy and 73.94 ± 6.50% F1 for valence, and 79.57 ± 5.99% accuracy and 74.89 ± 7.09% F1 for arousal. The decrease from subject-dependence is expected because DEAP contains strong inter-subject differences in baseline activity, signal amplitude, and emotional response patterns. Even so, the model retains reasonable performance under subject-independence, especially in arousal classification, indicating that the fused LSE and GSRE representations, together with temporal dynamics encoded by the TDE, contain information that can transfer beyond a single participant.

### 3.3. Experiment on SEED

Under subject-dependence evaluation, GLSTNet obtains 92.48 ± 3.30% accuracy and 92.43 ± 3.30% F1 on SEED. This result evaluates the model in a three-class setting with negative, neutral, and positive emotional states. The following subject-level table further reports participant-wise accuracy and F1 values to inspect individual-level behavior.

Table 4 reports the subject-dependent accuracy and F1 on the SEED dataset. The subject-dependent accuracy and F1 values indicate that most subjects maintain high recognition performance despite the three-class label space. The mean accuracy and F1 differ by only 0.05 percentage points, indicating that the model does not mainly favor one emotional category. This balance is important for SEED because neutral EEG responses can share patterns with both negative and positive states.

**Figure 13:**
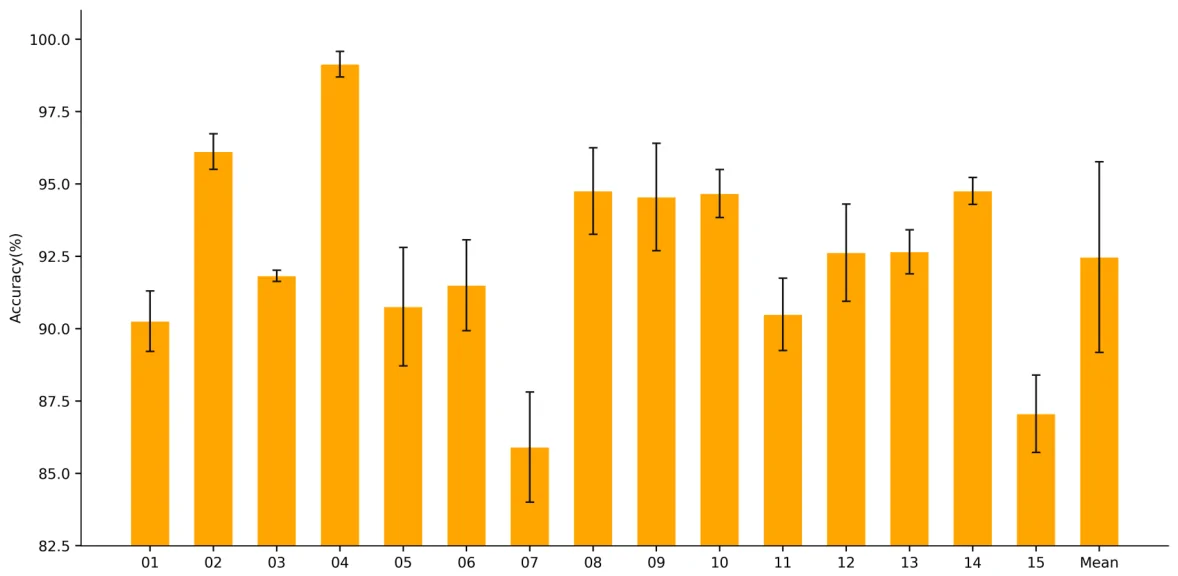
Subject-dependent accuracy on the SEED dataset. Orange bars represent the subject-wise accuracy, and black error bars represent the standard deviation across folds.

Figure 13 displays the subject-dependent accuracy on the SEED dataset. The accuracy plot indicates that most participants remain above 90.00%, while a small number of subjects have lower values. This distribution suggests that GLSTNet keeps strong performance for most participants, but subject-specific EEG differences still affect several cases.

**Figure 14:**
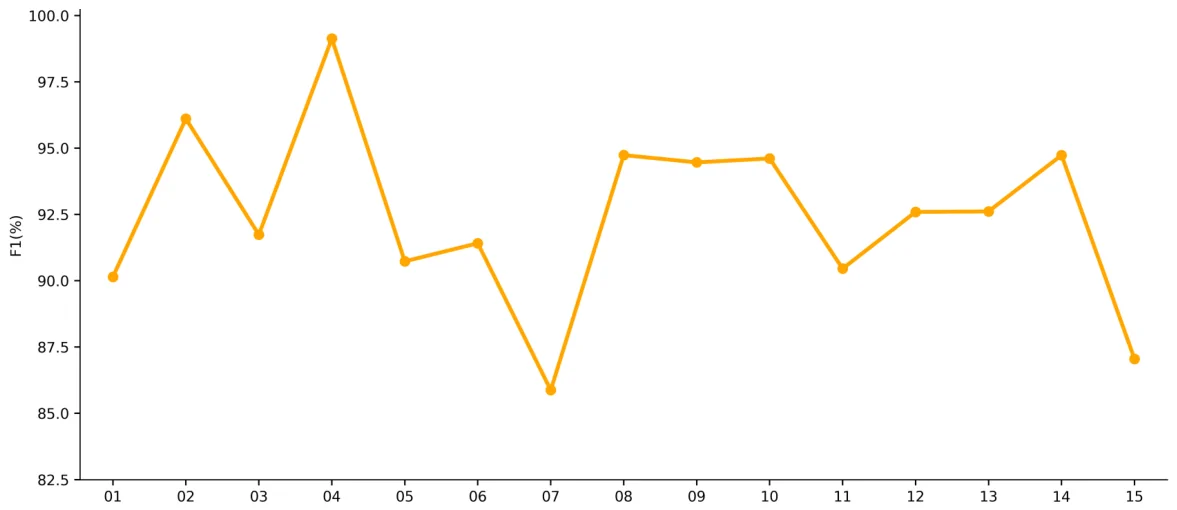
Subject-dependent F1 on the SEED dataset. The orange line and circular markers represent the subject-wise F1 values.

The subject-dependent F1 on the SEED dataset is plotted in Figure 14, which further examines whether the class-wise balance in Table 4 is preserved across participants. The F1 trend is close to the accuracy trend across subjects, which supports the observation that the model does not obtain high accuracy by over-predicting a single class. The similar behavior of accuracy and F1 also suggests balanced recognition among negative, neutral, and positive categories.

**Figure 15:**
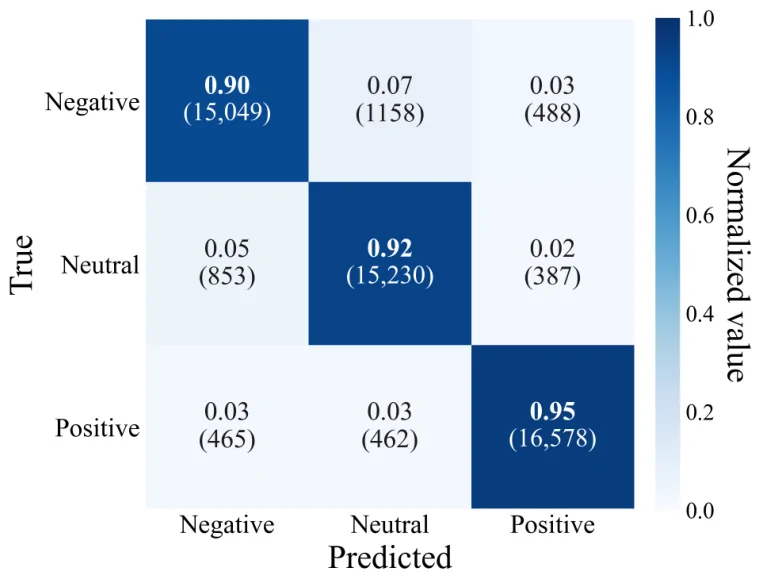
Confusion matrix on the SEED dataset.

Figure 15 presents the confusion matrix on the SEED dataset and identifies where the remaining errors mainly occur. Positive samples achieve the highest recall of 95.00%, followed by neutral samples with 92.00% and negative samples with 90.00%. Most misclassifications appear between negative and neutral states, suggesting that these categories have more similar EEG patterns than positive states. Nevertheless, the clear diagonal dominance indicates that the proposed model forms effective class-discriminative representations for three-class emotion recognition.

The subject-independence experiment on SEED evaluates the same model under a target-subject calibration setting. GLSTNet obtains 88.22 ± 4.70% accuracy and 88.14 ± 4.72% F1. Compared with the subject-dependence result, the performance drop is smaller than that observed on DEAP, indicating that the learned representation transfers more smoothly in this setting. The close accuracy and F1 values also show that the model does not achieve performance under subject-independence by over-predicting one emotional category. Taken together, the DEAP and SEED results indicate that subject-independence evaluation remains more affected by participant variability than subject-dependence evaluation, while GLSTNet still preserves transferable representations after target-subject calibration.

### 3.4. Comparison with Representative Methods

Table 5 compares the accuracy of GLSTNet with representative methods on the DEAP and SEED datasets. All baseline values are drawn from published subject-dependent evaluations rather than a unified reimplementation. The following descriptions identify the model structure, input representation, and dataset coverage relevant to the comparison.

(1) EEGNet uses temporal and depthwise separable convolutions for compact EEG representation learning; the comparison includes its DEAP valence and arousal results.
(2) DGCNN models dynamic electrode relations through graph convolution with spectral EEG features; results are available for both DEAP and SEED.
(3) MS-TimesNet combines multi-scale convolution and TimesNet with DE feature maps constructed from 0.5 s EEG windows; the comparison includes its DEAP results.
(4) BiDANN organizes DE representations according to hemispheric information and applies domain-adversarial learning; the comparison includes its SEED result.
(5) DGAT combines DE features, dynamic adjacency learning, and multi-head graph attention; results are available for both datasets.

Differences in experimental implementation can affect the reported performance even within subject-dependent evaluation. Window length controls temporal granularity and the number of samples in each segment, while sampling rate changes the number of samples available in a fixed-duration window and may influence filtering as well as DE and PSD estimation. Feature representation determines whether the classifier receives raw temporal samples, spectral descriptors, spatial maps, or graph inputs. Cross-validation implementation can also alter the composition of the training and test sets through fold construction and random partitioning. The published values are therefore used to contextualize GLSTNet under the reported settings.

GLSTNet ranks among the stronger results on both datasets. On DEAP, it outperforms DGCNN and MS-TimesNet in valence and arousal classification; compared with DGAT, it achieves higher arousal accuracy and slightly lower valence accuracy. On SEED, GLSTNet performs better than DGCNN and BiDANN but remains below DGAT. The comparison therefore places GLSTNet competitively across binary and three-class emotion recognition tasks, while the Discussion further examines the architectural differences underlying these results.

### 3.5. Ablation Study

Ablation experiments examine how each component affects recognition performance. Presented in Table 6 are the ablation results on the DEAP dataset. The full model achieves the best performance in both affective dimensions, showing that the complete architecture is more effective than its simplified variants.

Among the DEAP variants, removing the GSRE, temporal attention or the gated fusion mechanism causes smaller but consistent drops, indicating that these components provide incremental gains rather than acting as the sole drivers of performance. The full model remains the most stable configuration across both accuracy and F1. Presented in Table 7 are the ablation results on the SEED dataset, which further examine whether the same component-level pattern holds in the three-class setting.

The SEED ablation results show a different component-sensitivity pattern. Removing the GSRE causes only a small decrease, while removing temporal attention reduces accuracy by 0.90 percentage points. The larger drop after removing the gated fusion mechanism, from 92.48% to 90.43%, indicates that adaptive integration between LSE and GSRE representations is particularly important in the three-class setting.

### 3.6. Sensitivity Analysis

The sequence length *Q* controls the number of consecutive EEG windows encoded by the TDE. The main experiments use Q=6 as a unified default setting to keep the temporal input length consistent across datasets and tasks. Under subject-dependence evaluation, a smaller *Q* provides a shorter temporal context, whereas a larger *Q* allows the GRU and temporal attention mechanism to observe longer temporal dependencies. However, longer sequences may also introduce redundant or less stable segments. Therefore, the sensitivity analysis evaluates Q=2, Q=4, and Q=6 to examine the effect of temporal context length, while keeping the other experimental settings unchanged. Table 8 summarizes the sensitivity analysis of the sequence length *Q* under subject-dependence evaluation.

The sequence-length sensitivity results show task-dependent preferences. This analysis is intended to reveal how temporal context length affects different datasets and tasks, rather than to select a separate optimal *Q* for each task. On DEAP, Q=6 achieves the highest accuracy for valence, whereas Q=4 achieves the highest accuracy for arousal and Q=6 remains close to this value. On SEED, the shorter sequence length Q=2 achieves the highest accuracy. These observations indicate that the optimal temporal length is dataset- and task-dependent. A longer sequence can provide richer temporal context, but it may also introduce redundant or less stable windows. Therefore, Q=6 is retained as the unified default setting in the main experiments to keep the temporal input length consistent across datasets and tasks.

### 3.7. Visualization Analysis

**Figure 16:**
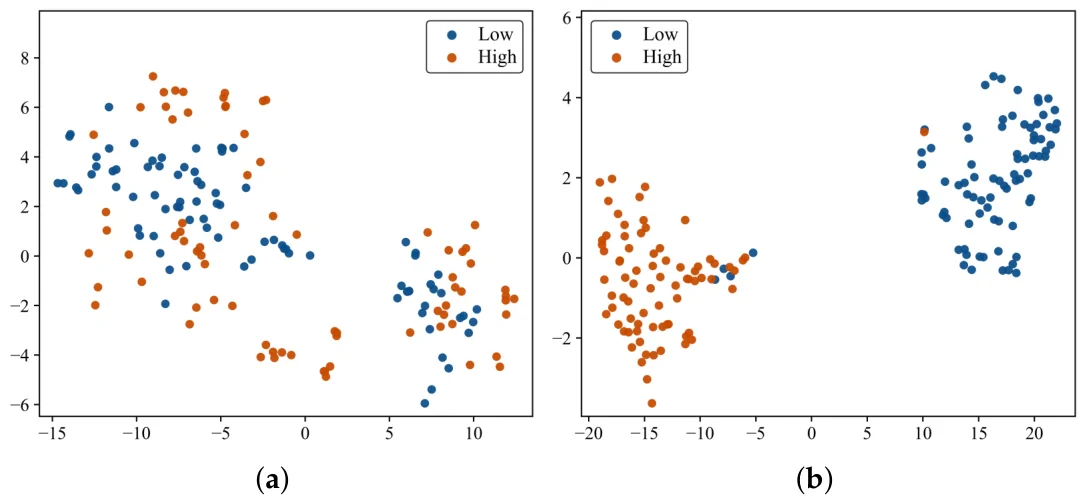
t-SNE visualization for DEAP valence. (**a**) Before feature learning. (**b**) After feature learning.

t-SNE visualization is used to inspect how the feature space changes after representation learning. The t-SNE visualization for DEAP valence before and after feature learning is presented in Figure 16. The original features are strongly intermixed, with no clear boundary between low- and high-valence samples. After feature learning, the two classes become more compact and easier to distinguish. This shift indicates that the learned representations separate the two valence classes more clearly than the original features.

**Figure 17:**
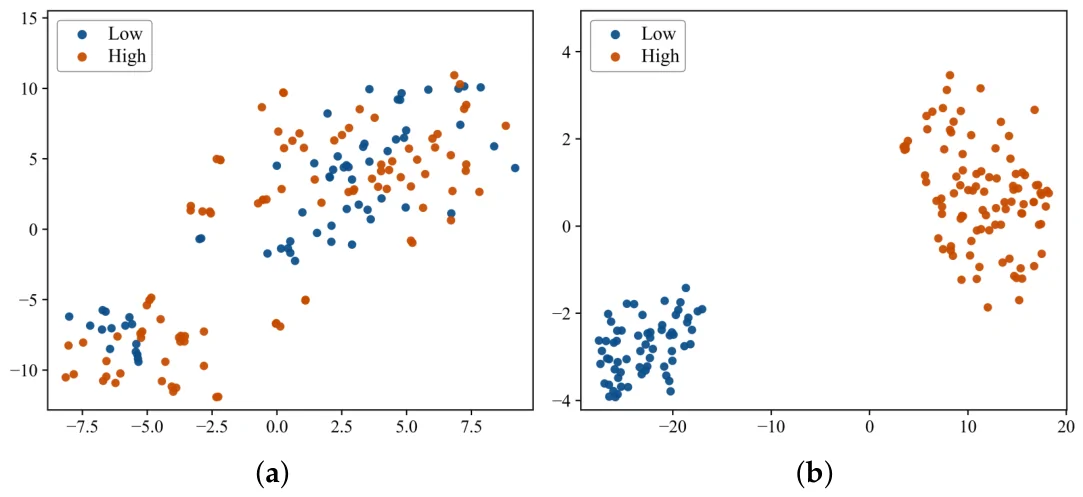
t-SNE visualization for DEAP arousal. (**a**) Before feature learning. (**b**) After feature learning.

Presented in Figure 17 is the t-SNE visualization for DEAP arousal before and after feature learning. The low- and high-arousal samples are entangled before feature learning, whereas the learned features form more concentrated class-wise regions. This pattern is consistent with the high arousal accuracy and indicates clearer class separation after GLSTNet representation learning.

**Figure 18:**
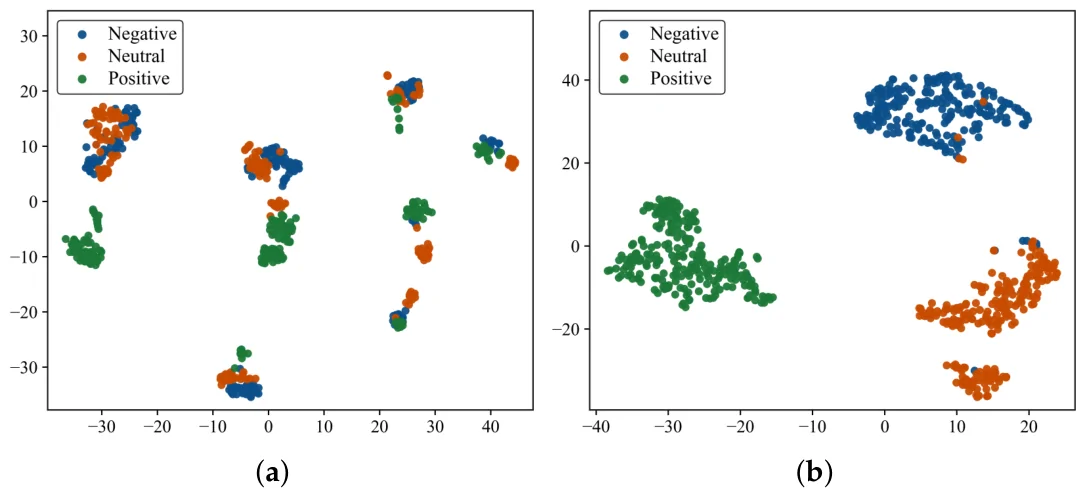
t-SNE visualization for SEED. (**a**) Before feature learning. (**b**) After feature learning.

Figure 18 presents the t-SNE visualization for SEED before and after feature learning in the three-class setting. The original samples are largely mixed, making negative, neutral, and positive states difficult to separate in the input feature space. After feature learning, the three categories form more organized class-wise distributions and clearer cluster boundaries. Some overlap remains, but the overall separation is improved, which is consistent with the high three-class accuracy on SEED. The visualization is consistent with the quantitative results and reveals clearer class-wise structure after representation learning.

**Figure 19:**
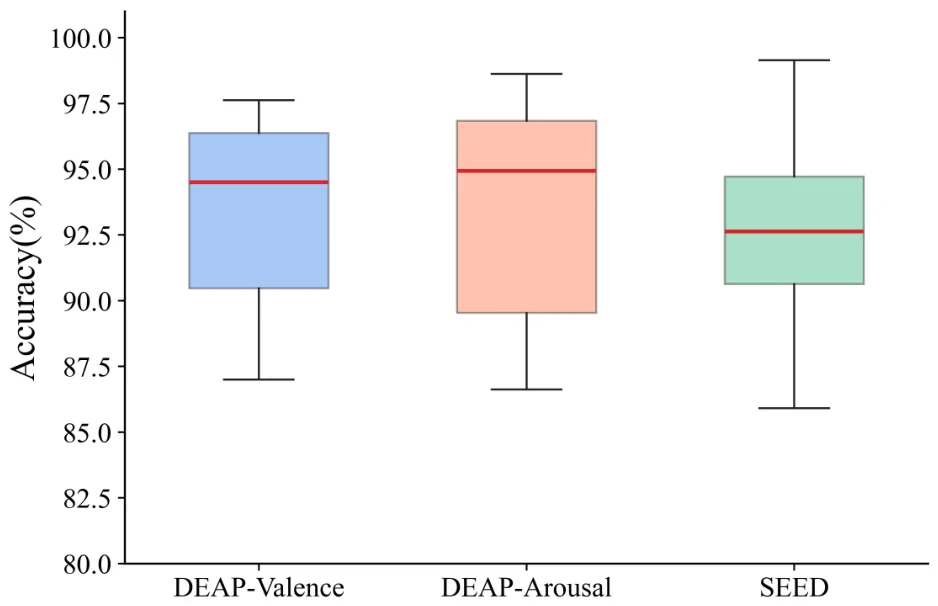
Subject-dependent accuracy distributions on DEAP and SEED.

Figure 19 summarizes the subject-dependent accuracy distributions on DEAP and SEED. DEAP valence and arousal have higher median accuracy values of 94.50% and 94.94%, respectively, while SEED has a slightly lower median accuracy of 92.63%. The interquartile range of SEED is the smallest among the three tasks, indicating that the middle 50% of participants have relatively concentrated performance. However, SEED also has the largest overall range, from 85.91% to 99.14%, suggesting that a few participants deviate more strongly from the central distribution. This pattern indicates that the proposed model maintains stable performance for most participants on SEED, while individual differences still affect a small number of subjects.

## 4. Discussion

### 4.1. Relation to Existing EEG Emotion Recognition Methods

Compact convolutional networks such as EEGNet provide efficient local filtering and spatial–spectral representation learning. Graph-based models such as DGCNN and DGAT are better suited to describing dependencies between distant electrodes. Temporal–spatial models such as MS-TimesNet focus on temporal organization across EEG segments. These designs each capture an important part of EEG emotion responses, although local scalp patterns, long-range electrode relations, and temporal evolution are often handled separately.

GLSTNet brings these elements into a unified framework. The LSE extracts local spatial and spatial–spectral information from compact maps, the GSRE models global electrode relations through an adaptive graph, gated fusion adjusts the contribution of the two spatial representations at each time slice, and the TDE encodes sequence-level temporal dynamics. The comparison with representative methods is therefore consistent with the design motivation of GLSTNet, since the improvement comes from jointly modeling local scalp patterns, non-adjacent electrode relations, and temporal changes in EEG responses.

### 4.2. Sampling Frequency

Sampling frequency enters the pipeline before the GLSTNet input is assembled. With the same 0.5 s window length, a 128 Hz signal provides 64 sampling points per window, whereas a 200 Hz signal provides 100 sampling points. This difference may affect band-pass filtering and the estimation of DE and PSD features from short EEG segments. Once the features have been extracted, each window is represented as an electrode-wise band feature matrix and mapped to the same 8×9 compact spatial map. Sampling frequency therefore changes the descriptor values more directly than the input shape or the network architecture.

SEED is further evaluated after resampling from 200 Hz to 128 Hz to examine this preprocessing factor. The window length, frequency bands, feature configuration, subject-dependence setting, and model hyperparameters are kept unchanged, allowing the comparison to focus on the sampling rate used before feature extraction. Table 9 reports the SEED results under the original and resampled settings.

After resampling SEED from 200 Hz to 128 Hz, accuracy changes from 92.48% to 91.53%, and F1 changes from 92.43% to 91.48%. The decrease is about 0.95 percentage points for both metrics. The result shows that sampling frequency can influence performance through spectral feature estimation, but the effect remains modest when the same frequency bands and window length are preserved. Differences in electrode montage, label space, stimuli, preprocessing pipelines, and subject variability may also contribute to the performance differences observed across datasets.

### 4.3. Spectral Feature Effects

The ablation study evaluates architectural components, whereas the spectral-feature experiments address the role of hand-crafted descriptors in the observed performance. Table 10 summarizes the effects of DE, PSD, and DE+PSD on DEAP and SEED under the subject-dependence setting.

For GLSTNet, DE+PSD performs best on DEAP, indicating that PSD provides complementary power-related information for valence and arousal classification. On SEED, DE achieves the highest accuracy and F1 with the lowest variability, whereas PSD and DE+PSD lead to lower performance and larger fluctuations. Thus, the preferred spectral input depends on dataset characteristics rather than being fixed across tasks.

To examine whether the gain comes only from these descriptors, SVM, MLP, EEGNet, and DGCNN are evaluated with matched DE, PSD, or DE+PSD inputs using the same preprocessed samples and subject-wise partitions. The comparison covers flattened feature classification, convolutional learning, and graph-based representation learning. Table 11 presents the corresponding DEAP results.

On DEAP, DE+PSD also yields the highest accuracy for SVM, EEGNet, and DGCNN, in line with the GLSTNet configuration. Even under this matched input setting, EEGNet, the strongest comparison model, reaches 90.36 ± 4.37% accuracy for valence and 91.13 ± 3.88% for arousal, whereas GLSTNet reaches 93.50 ± 3.22% and 93.79 ± 3.64%, respectively. The remaining gap indicates that the architecture still contributes beyond the choice of DE+PSD features. Table 12 presents the corresponding SEED comparison.

On SEED, DE is also the preferred input for MLP, EEGNet, DGCNN, and GLSTNet, so the weaker PSD and DE+PSD results are not specific to the proposed model. EEGNet again provides the strongest comparison, with 90.35 ± 3.55% accuracy and 90.29 ± 3.56% F1, while GLSTNet obtains 92.48 ± 3.30% and 92.43 ± 3.30% under the same input and partitions.

Overall, DE and PSD provide useful spectral cues, but they do not by themselves explain the performance of GLSTNet. The advantage retained under matched feature configurations is more closely related to the way GLSTNet combines compact spatial maps, adaptive electrode relations, gated spatial fusion, and temporal dynamics encoding.

### 4.4. Computational Feasibility for Cloud-Side Analysis

The workflow in Figure 1 is intended for cloud-side EEG analysis rather than direct real-time inference on a wearable device. Wearable sensors acquire EEG signals, the mobile terminal supports signal display and upload, and GLSTNet performs analysis on the cloud side. Table 13 summarizes the computational profile of GLSTNet under the main DEAP and SEED configurations.

The computational profile is measured on an NVIDIA GeForce RTX 4090 GPU (NVIDIA Corporation, Santa Clara, CA, USA). Prediction time in Table 13 denotes the time required to classify one prepared sequence-level GLSTNet input composed of *Q* consecutive EEG windows. The timer starts from the prepared model input and ends at the class-probability prediction. These measurements describe model-side prediction in the intended cloud-side analysis setting, where computation is performed after signal upload.

### 4.5. Limitations and Future Work

Several aspects can be further refined in future work. GLSTNet currently relies on separately computed spectral features, including DE and PSD, before model training. An end-to-end extension that learns from EEG signals directly, or jointly optimizes spectral filtering with spatial–temporal representation learning, may reduce the need for manual feature preparation while keeping the discriminative structure of the current framework.

The present workflow is more suitable for cloud-side EEG analysis after signal upload than for direct inference on wearable devices. Although the measured prediction time is short for a prepared model input, practical response time also depends on signal acquisition, data transmission, feature extraction, and system integration. Future work will therefore examine system-level optimization, model compression, and validation on application-oriented or clinical EEG recordings to improve the practical use of GLSTNet in EEG-based biosensing.

## 5. Conclusions

This study presents GLSTNet, a global-local spatial relations and temporal dynamics network for EEG-based emotion recognition. The framework extracts multi-band EEG features and arranges them into compact spatial maps. The LSE learns local spatial and spatial–spectral representations, the GSRE models global spatial relations through an adaptive electrode graph, the gated fusion mechanism integrates local and global representations, and the TDE encodes temporal dynamics across consecutive EEG windows. Under subject-dependence, GLSTNet achieves 93.50% accuracy for DEAP valence, 93.79% accuracy for DEAP arousal, and 92.48% accuracy on SEED. Under subject-independence with target-subject calibration, it obtains 75.61% accuracy for DEAP valence, 79.57% accuracy for DEAP arousal, and 88.22% accuracy on SEED. Further evidence from representative-method comparison, component ablation, and feature-space visualization confirms that GLSTNet benefits from the joint modeling of local spatial patterns, global electrode relations, and temporal dynamics. Spectral-feature and feature-only analyses also show that although DE and PSD provide informative EEG descriptors, the advantage of GLSTNet is closely related to how these descriptors are organized into spatial maps and modeled across electrodes and time windows. Practical feasibility is further supported by the SEED resampling experiment and the computational profile. Resampling from 200 Hz to 128 Hz leads to only a modest performance change under the same window length and frequency-band setting, and GLSTNet completes model-side prediction within several milliseconds for a prepared sequence-level input in the cloud-side analysis setting. These findings support the use of global-local spatial relations and temporal dynamics modeling for EEG-based biosensing, while future work will further explore end-to-end feature learning and practical system-level optimization.

## Figures and Tables

**Table 1:** Summary of the DEAP and SEED datasets used in this work.

Item	DEAP	SEED
Subjects	32	15
EEG channels	32	62
Sampling rate	128 Hz	200 Hz
Stimuli	40 music videos	15 film clips/session
Sessions	1	3
Emotion labels	Valence, arousal	Positive, neutral, negative
Task type	Binary classification	Three-class classification

**Table 2:** Subject-dependent accuracy (%) on the DEAP dataset.

Subject	Valence	Arousal	Subject	Valence	Arousal
01	97.25	98.62	17	90.50	88.75
02	89.00	89.25	18	96.88	97.25
03	93.50	96.25	19	96.12	94.62
04	91.25	89.62	20	97.62	97.12
05	88.75	90.88	21	95.38	96.25
06	96.62	95.25	22	90.75	89.12
07	96.75	97.88	23	97.12	97.25
08	96.38	96.00	24	89.88	95.50
09	89.62	89.00	25	94.62	98.50
10	95.62	97.38	26	91.88	89.12
11	87.00	86.62	27	96.12	96.75
12	94.25	95.62	28	90.38	92.25
13	95.50	96.00	29	95.75	94.50
14	92.12	94.25	30	88.25	88.88
15	96.38	94.50	31	94.38	93.50
16	97.12	97.62	32	89.38	87.25

**Table 3:** Subject-dependent F1 (%) on the DEAP dataset.

Subject	Valence	Arousal	Subject	Valence	Arousal
01	97.24	98.57	17	90.33	88.05
02	88.86	88.74	18	96.72	97.07
03	93.43	93.82	19	96.03	93.77
04	90.77	89.11	20	97.56	95.66
05	88.21	90.83	21	95.36	93.99
06	95.47	95.11	22	90.61	88.58
07	96.09	97.73	23	96.81	96.27
08	96.34	95.91	24	89.78	91.80
09	89.62	88.40	25	94.60	98.10
10	95.62	97.35	26	90.90	88.77
11	86.27	85.62	27	94.70	96.28
12	94.24	92.11	28	89.67	92.13
13	95.37	91.19	29	95.63	94.12
14	92.12	93.25	30	86.38	88.84
15	96.37	94.48	31	94.25	93.48
16	96.89	97.62	32	89.35	85.35

**Table 4:** Subject-dependent accuracy (%) and F1 (%) on the SEED dataset.

Subject	Accuracy	F1	Subject	Accuracy	F1
01	90.26	90.15	09	94.55	94.46
02	96.12	96.11	10	94.67	94.61
03	91.83	91.73	11	90.50	90.46
04	99.14	99.13	12	92.63	92.59
05	90.76	90.73	13	92.66	92.61
06	91.50	91.41	14	94.76	94.73
07	85.91	85.87	15	87.06	87.05
08	94.76	94.74			

**Table 5:** Accuracy (%) comparison with representative methods on the DEAP and SEED datasets.

Method	DEAP Valence	DEAP Arousal	SEED
EEGNet [31]	82.31	85.00	–
DGCNN [18]	91.82	91.93	90.40
MS-TimesNet [32]	90.45	91.31	–
BiDANN [33]	–	–	92.38
DGAT [34]	93.55	93.19	94.00
GLSTNet (ours)	93.50	93.79	92.48

**Table 6:** Ablation results on the DEAP dataset. Values are reported as mean ± std (%).

Variant	Valence		Arousal	
	Accuracy (%)	F1 (%)	Accuracy (%)	F1 (%)
Full model	93.50 ± 3.22	93.18 ± 3.36	93.79 ± 3.64	92.88 ± 3.71
w/o GSRE	93.15 ± 3.57	92.77 ± 3.74	93.69 ± 3.41	92.69 ± 3.53
w/o temporal attention	93.14 ± 3.24	92.80 ± 3.31	93.71 ± 3.27	92.69 ± 3.47
w/o gated fusion	93.05 ± 3.59	92.73 ± 3.67	93.73 ± 3.64	92.76 ± 3.84

**Table 7:** Ablation results on the SEED dataset. Values are reported as mean ± std (%).

Variant	Accuracy (%)	F1 (%)
Full model	92.48 ± 3.30	92.43 ± 3.30
w/o GSRE	92.35 ± 3.72	92.29 ± 3.76
w/o temporal attention	91.58 ± 6.61	91.55 ± 6.61
w/o gated fusion	90.43 ± 8.50	90.37 ± 8.49

**Table 8:** Sensitivity analysis of the sequence length *Q* under subject-dependence evaluation. Values are reported as mean ± std (%).

Task	Q=2	Q=4	Q=6
DEAP Valence	92.74 ± 3.25	93.32 ± 3.38	93.50 ± 3.22
DEAP Arousal	93.64 ± 2.84	94.06 ± 2.96	93.79 ± 3.64
SEED	93.42 ± 2.99	92.99 ± 3.34	92.48 ± 3.30

**Table 9:** Effect of SEED resampling on subject-dependence evaluation. Values are reported as mean ± std (%).

Sampling Rate	Accuracy (%)	F1 (%)
200 Hz	92.48 ± 3.30	92.43 ± 3.30
128 Hz	91.53 ± 4.85	91.48 ± 4.87

**Table 10:** Effects of spectral feature configurations on DEAP and SEED under subject-dependence evaluation. Values are reported as mean ± std (%).

Task	Feature	Accuracy (%)	F1 (%)
	DE	90.61 ± 4.22	90.08 ± 4.47
DEAP Valence	PSD	90.40 ± 9.51	90.03 ± 9.73
	DE+PSD	93.50 ± 3.22	93.18 ± 3.36
	DE	91.90 ± 3.62	90.55 ± 3.74
DEAP Arousal	PSD	90.02 ± 9.91	88.66 ± 10.54
	DE+PSD	93.79 ± 3.64	92.88 ± 3.71
	DE	92.48 ± 3.30	92.43 ± 3.30
SEED	PSD	81.93 ± 11.17	81.67 ± 11.74
	DE+PSD	82.63 ± 13.07	81.98 ± 14.31

**Table 11:** Results of comparison models with different spectral features on the DEAP dataset under subject-dependence evaluation. Values are reported as mean ± std (%).

Model	Feature	Valence Acc.	Valence F1	Arousal Acc.	Arousal F1
	DE	84.24 ± 4.38	83.56 ± 4.35	84.34 ± 4.41	82.34 ± 3.71
SVM	PSD	82.36 ± 6.02	81.68 ± 5.97	82.55 ± 6.55	80.47 ± 6.25
	DE+PSD	86.91 ± 4.91	86.38 ± 4.96	87.04 ± 4.45	85.47 ± 4.04
	DE	83.25 ± 6.03	82.25 ± 6.37	84.74 ± 5.18	81.67 ± 5.63
MLP	PSD	75.64 ± 9.56	74.03 ± 9.81	78.29 ± 8.89	72.68 ± 10.41
	DE+PSD	84.58 ± 5.89	83.75 ± 6.00	85.63 ± 6.00	83.01 ± 6.27
	DE	89.82 ± 4.51	89.36 ± 4.61	90.89 ± 3.91	89.75 ± 3.67
EEGNet	PSD	80.02 ± 10.72	78.10 ± 12.39	82.50 ± 9.49	79.11 ± 10.92
	DE+PSD	90.36 ± 4.37	89.95 ± 4.47	91.13 ± 3.88	90.05 ± 3.58
	DE	68.92 ± 6.74	66.83 ± 6.55	70.38 ± 7.16	64.89 ± 4.59
DGCNN	PSD	63.89 ± 7.85	59.78 ± 7.74	68.24 ± 8.13	59.12 ± 5.97
	DE+PSD	69.49 ± 6.22	67.42 ± 5.93	71.33 ± 6.67	66.14 ± 5.19

**Table 12:** Results of comparison models with different spectral features on the SEED dataset under subject-dependence evaluation. Values are reported as mean ± std (%).

Model	Feature	Accuracy (%)	F1 (%)
	DE	83.40 ± 6.26	83.28 ± 6.29
SVM	PSD	77.98 ± 5.64	77.89 ± 5.65
	DE+PSD	84.23 ± 5.31	84.13 ± 5.32
	DE	85.98 ± 4.59	85.89 ± 4.61
MLP	PSD	79.20 ± 5.80	79.10 ± 5.81
	DE+PSD	85.46 ± 4.35	85.38 ± 4.36
	DE	90.35 ± 3.55	90.29 ± 3.56
EEGNet	PSD	56.93 ± 15.27	50.83 ± 20.06
	DE+PSD	58.29 ± 16.89	51.67 ± 21.85
	DE	63.83 ± 7.76	62.50 ± 8.23
DGCNN	PSD	57.70 ± 9.20	56.99 ± 9.44
	DE+PSD	60.95 ± 7.94	60.23 ± 8.00

**Table 13:** Computational profile of GLSTNet under the main DEAP and SEED configurations.

Dataset	Parameters (M)	FLOPs (G)	Prediction Time (ms)
DEAP	2.02	0.608	4.71
SEED	2.02	0.605	4.73

## Data Availability

The DEAP dataset is available from its original provider at https://www.eecs.qmul.ac.uk/mmv/datasets/deap/ (accessed on 4 April 2026). The SEED dataset is available from the BCMI Laboratory at Shanghai Jiao Tong University at https://bcmi.sjtu.edu.cn/~seed/ (accessed on 4 April 2026). These datasets are subject to the access policies of their respective providers. The code used in this study will be made available from the corresponding author upon reasonable request.
